# Venovenous Extracorporeal Membrane Oxygenation for Alveolar Hemorrhage During Mitral Valve Repair via Right Minithoracotomy in a Patient With Myelodysplastic Syndrome

**DOI:** 10.7759/cureus.79320

**Published:** 2025-02-19

**Authors:** Motoki Nagatsuka, Tohru Asai, Kenichiro Noguchi, Daisuke Hama, Chishio Arai

**Affiliations:** 1 Department of Cardiovascular Surgery, Shonan Kamakura General Hospital, Kamakura, JPN

**Keywords:** focal alveolar hemorrhage, minimally invasive cardiac surgical procedures, mitral valve surgery, myelodysplastic syndrome (mds), venovenous extracorporeal membrane oxygenation

## Abstract

Cardiac surgery in patients with myelodysplastic syndrome (MDS) is difficult because extracorporeal circulation increases the risk of infection and hemorrhage. We discuss the efficacy of venovenous extracorporeal membrane oxygenation (VV-ECMO) in managing intraoperative alveolar hemorrhage in a patient with MDS undergoing minimally invasive cardiac surgery (MICS) for mitral valve repair. A 76-year-old man with MDS underwent mitral valve repair via right minithoracotomy with standard cardiopulmonary bypass. Left lung hemorrhage developed after declamping of the aorta, and oxygenation support was necessary when the cardiopulmonary bypass was finished, as a bridge until hemostasis. VV-ECMO was established, which involves withdrawing blood from the right femoral vein and returning it via the right internal jugular vein. Platelet transfusion and protamine were administered. Hemostasis was achieved, and the patient was admitted to the intensive care unit without mechanical circulatory support. VV-ECMO proved successful for MICS with hemorrhagic pulmonary complications.

## Introduction

Myelodysplastic syndrome (MDS) represents a series of oligoclonal disorders of hematopoietic stem cells characterized by hematopoietic failure, clinically manifested as anemia of varying severity, neutropenia, and/or thrombocytopenia [1]. Cardiac surgery in patients with MDS is difficult because extracorporeal circulation increases the risk of infection and hemorrhage. There have been several reports of hemorrhagic complications after cardiac surgery in patients with MDS, but there are no reports of alveolar hemorrhage [2]. Although minimally invasive cardiac surgery (MICS) is known to have a lower risk of postoperative bleeding and infection due to the absence of a median sternotomy [3], the specific complications of MICS due to isolated lung ventilation and a narrow surgical field should also be considered. A case of left alveolar hemorrhage during MICS mitral valve repair, caused by the hemorrhagic tendency of MDS and difficulty in maintaining oxygenation during isolated lung ventilation, was converted from extracorporeal circulation to venovenous extracorporeal membrane oxygenation (VV-ECMO). After the introduction of VV-ECMO, heparin neutralization with protamine was promptly administered and hemostasis was achieved with platelet transfusion. We discuss the efficacy of VV-ECMO for intraoperative alveolar hemorrhage in a patient with MDS for MICS mitral valve repair.

## Case presentation

A 76-year-old man was receiving azacitidine and red blood cell transfusions every two weeks in the hematology department due to MDS. He underwent MICS mitral valve repair for mitral regurgitation due to P3 prolapse, which was diagnosed after exacerbation of dyspnea on exertion. Preoperative laboratory tests showed no leukocytopenia, thrombocytopenia, or coagulopathy other than anemia (Table 1), and granulocyte-colony stimulating factor products or platelet transfusions were not administered. 

**Table 1:** Investigation profile of the patient at the time of admission. PT-INR, prothrombin time-international normalized ratio

Investigations	Patient	Reference values
Leukocytes	33×10^2^/μL	33-86×10^2^/μL
Hemoglobin	7.8 g/dL	13.7-16.8 g/dL
Platelets	15.8×10^4^/μL	15.8-34.8×10^4^/μL
PT-INR	1.25	0.89-1.12
Fibrinogen	244.8 mg/dL	150-400 mg/dL
Antithrombin-III	95.8%	75-125%

**Figure 1:**
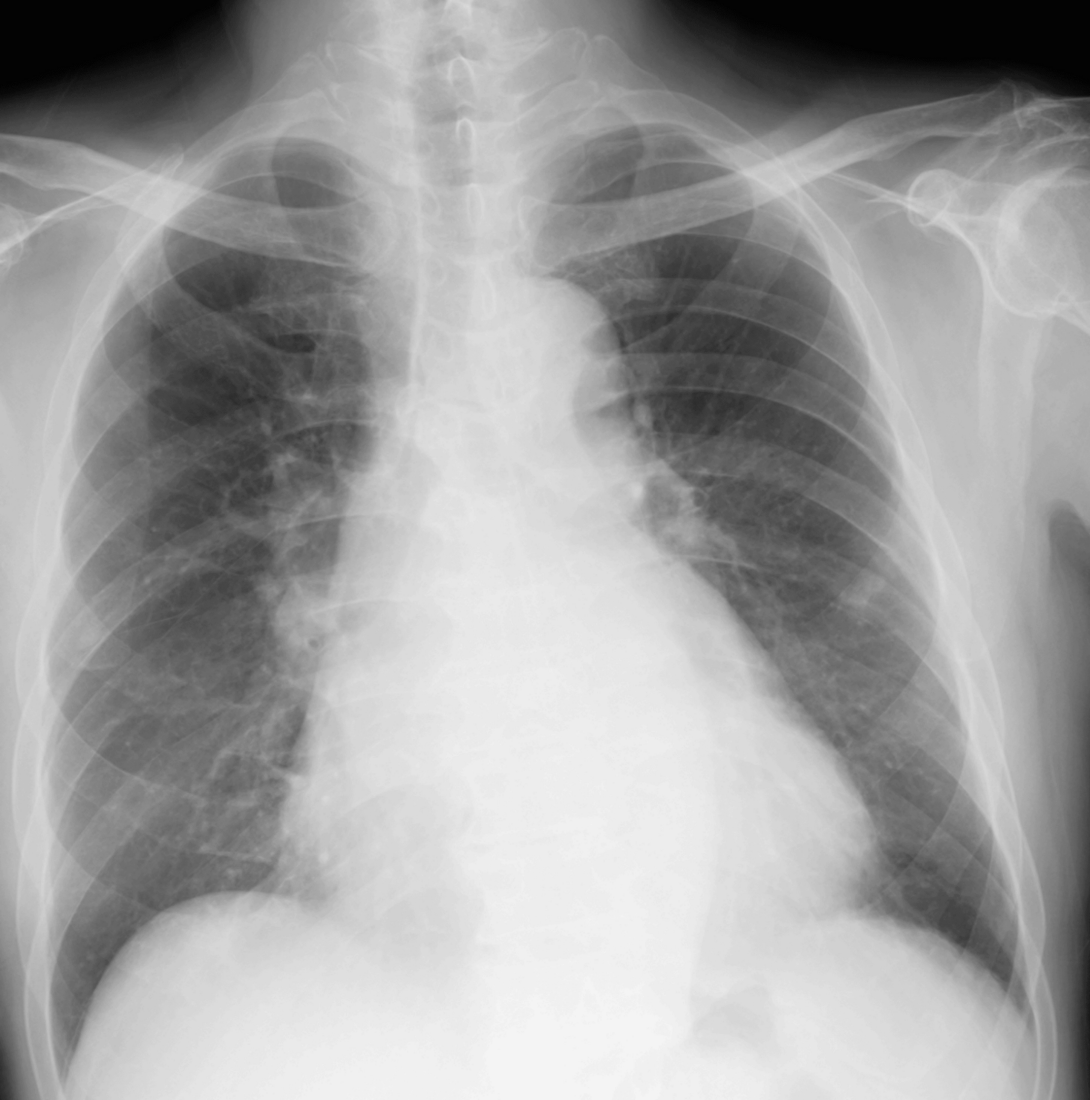
Preoperative AP chest radiograph. AP, anteriorposterior

The X-ray of the patient showed no ground-glass opacities in the bilateral lungs (Figure 1).

**Figure 2:**
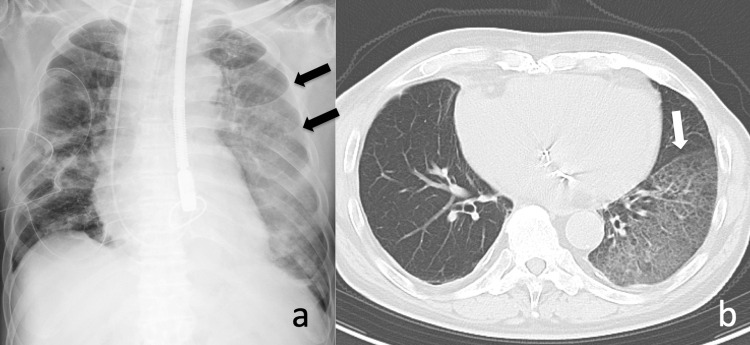
Postoperative images. a) Radiograph of the chest shows ground-glass opacities in the left lung on postoperative day 0. b) Postoperative plain computed tomography of the chest shows ground-glass opacities in the entire left lower lobe, interpreted as alveolar hemorrhage.

The patient was approached through a right mini-thoracotomy in the right 4th intercostal space. Extracorporeal circulation was established by withdrawing blood from the right internal jugular vein and the right femoral vein, returning the blood via the left axillary artery. The operative procedure was performed with two artificial chordae and semi-rigid full rings (Physio II Annuloplasty ring 34 mm, Edwards Lifescience, Irvine, California, United States), and there was no residual mitral regurgitation. After declamping the ascending aorta, bleeding was observed from the left main bronchus, and alveolar hemorrhage was diagnosed (Figure 2).

Since it was difficult to maintain oxygenation, the patient was switched to VV-ECMO by withdrawing from the right femoral vein and returning via the right internal jugular vein, and protamine and platelet transfusions were administered. After hemostasis was achieved and oxygenation improved, the patient was weaned from the VV-ECMO (running time is 63 minutes), and his chest was closed. The patient was admitted to the intensive care unit with no mechanical circulatory support devices and stable hemodynamic status. 

On the sixth postoperative day, the patient was discharged from the hospital with good course.

## Discussion

MDS is recognized as a preleukemic disorder, and coagulopathy, with or without thrombocytopenia, is common in patients with MDS [4]. Platelet dysfunction is common in patients with MDS, even in those with a normal platelet count [5]. Perioperative bleeding risks are associated with MDS and the use of extracorporeal circulation. The patient exhibited a noticeable bleeding tendency during the operation, which required platelet transfusions to control coagulability. Cardiac surgery for patients with MDS is rare, only 14 cases have been reported, and only one MICS procedure for a patient with MDS has been reported to date [6]. Although cardiac tamponade and subarachnoid hemorrhage have been reported as bleeding complications in these patients, alveolar hemorrhage has not been reported. Mechanisms of airway hemorrhage include direct injury to the airways and blood vessels, dilation and congestion of bronchial veins, increased capillary permeability, abnormalities in the platelet and coagulation-fibrinolytic systems, and iatrogenic factors such as Swan-Ganz catheters. The alveolar hemorrhage in this case was left-sided, while the intubation tube and Swan-Ganz catheter were placed on the right side, so these were not considered the cause. In addition to abnormal platelet function due to MDS and a bleeding tendency from prolonged activated clotting time during heparinization, mechanical stimuli such as sputum aspiration may have triggered the bleeding. In other words, it was deemed essential to immediately reverse the heparinization and restore the coagulation system. Switching to VV-ECMO was appropriate because the patient was not in an oxygen state that would allow immediate weaning from extracorporeal circulation. The patient was easily switched to VV-ECMO after weaning from extracorporeal circulation by using two blood circuits, one from the right internal jugular vein and the other from the right femoral vein. This method has been reported to avoid thrombotic complications without anticoagulation therapy during VV-ECMO support for MICS mitral valve repair via a right mini-thoracotomy [6]. In this case, blood was drawn from the right femoral vein and returned via the right internal jugular vein, and VV-ECMO was initiated immediately, driven for 63 minutes, with no thrombotic complications observed. The usefulness of VV-ECMO without anticoagulation therapy for diffuse alveolar hemorrhage has been reported [7], but registry data for cases using ECMO in diffuse alveolar hemorrhage suggests a survival rate of 73%, indicating that it is a serious complication that must be addressed promptly and appropriately [8].

## Conclusions

MICS has multiple advantages compared to standard open-heart surgery: lower complication rates, reduced risk of infection, less blood loss, fewer arrhythmia events, shorter intensive care unit and hospital stays, and higher patient satisfaction due to improved cosmetic healing of the wound. However, MICS in patients with a high risk of bleeding, such as those with MDS, can lead to unexpected complications, such as alveolar hemorrhage during isolated pulmonary ventilation. This VV-ECMO technique appeared to be a safe treatment option for a patient who experienced impaired oxygenation due to a pulmonary complication during MICS mitral valve repair.
